# Not Seeing the Forest for the Trees: The Impact of Multiple Labelling on Consumer Choices for Olive Oil

**DOI:** 10.3390/foods9020186

**Published:** 2020-02-13

**Authors:** Luis Pérez y Pérez, Azucena Gracia, Jesús Barreiro-Hurlé

**Affiliations:** 1 Unidad de Economía Agroalimentaria y de los Recursos Naturales, Centro de Investigación y Tecnología Agroalimentaria de Aragón (CITA), Av Montañana, 930, 50059 Zaragoza, Spain; lperez@aragon.es; 2 Instituto Agroalimentario de Aragón (CITA-Universidad de Zaragoza), 50059 Zaragoza, Spain; 3 European Commission. Joint Research Centre (JRC), 41092 Seville, Spain; jesus.barreiro-hurle@ec.europa.eu

**Keywords:** European food quality labels, protected designation of origin (PDO), organic production, extra virgin olive oil (EVOO), Spain

## Abstract

Multiple quality labels that signal whether a particular food has special characteristics relating to geographical origin or production method have become standard within European food policy. The aim of this paper was to investigate how two of these labels in particular influence consumers’ food choices. We assessed consumers’ preferences for an extra virgin olive oil (EVOO) displaying EU quality labels and focus on whether they are complements or substitutes. In order to do so, we used a discrete choice experiment (DCE) to estimate main and two-way interactions effects with data from a self-administrated survey in a Spanish region. Results indicate that while consumers positively value both the Protected Designation of Origin (PDO) and the organic labels, the valuation for PDO is almost double that of the valuation of the organic label. Furthermore, the findings show that for a majority of consumers considered both labels substitutes, while a small group considered them complements. These findings can help producers identify an optimal labelling strategy to maximize returns on certification investments.

## 1. Introduction

Olive oil is one of the main components of the Mediterranean diet that is considered worldwide as one of the healthiest food diets. In recent years, consumption of olive oil in Mediterranean countries has shifted towards one of a higher quality. Quality olive oils can be differentiated using EU regulated labels such as those under regulation of a quality scheme for agricultural foodstuffs [1,2]. The first one regulates the designation of geographical origin and the second the organic production system.

Quality schemes for agricultural products and foodstuffs in the EU are laid down in [1]. This Regulation is an amending version based, *inter alia*, on [3] for wine products, [4] relating to honey, [5] for traditional specialties and [6] on the protection of geographical indications and designations of origin for agricultural products and foodstuffs.

Agricultural foodstuffs bearing the Protected Designation of Origin (PDO) label are those whose quality or characteristics are determined by the geographical environment with its natural and human factors, and whose production, processing and preparation are always carried out in the defined geographical area from which they take their name. Registration under a PDO guarantees compliance with quality requirements additional to those required for other products without this mention [1]. Registration is voluntary for the products that meet these requirements and they are included in an EU register. There are currently 102 PDOs registered in Spain and products labelled as PDOs are protected by intellectual property rights and can be recognized by the EU symbol on their packaging [7,8].

Organic production is an agri-food management and production system that combines best environmental practices and the application of high animal welfare standards that lead to high levels of biodiversity and the preservation of natural resources. Organic production has been regulated in Spain since 1989. With 2.2 million hectares of cultivated area in 2018, Spain occupies the first place in organic farming in the EU and is among the top five in the world. In 1993, the first Community Regulation came into effect and currently Regulation on organic production and labelling of organic products is in force [2]. All organic products packaged must bear the EU logo and the numerical code of the responsible inspection body, in addition to the producer’s own brand and the specific terms of organic production. For the purpose of our research, extra virgin olive oil (EVOO) was selected for two main reasons. Firstly, olive grove is one of Spain’s most important agricultural land use covering 2.5 million hectares and producing about 60% of the world’s olive oil. Secondly, this product has a high prevalence of geographic origin and organic labels.

EU labels were introduced to help consumers in making their food choice as the information they carry transforms the credence attributes, which cannot be observed by the consumers even after purchase, in our case, the geographical origin and the production method, into search attributes, which consumers can look for when purchasing. In addition, labels also provide a form of quality assurance in terms of product traceability to with a given production area [9]. However, different studies into the effect of labels on food purchasing decisions show that they are not the main drivers of consumer choice and that other product characteristics such as price, color, origin, etc. are more relevant. In the case of olive oil, [10,11] found that price is a more important driver than PDO certification. Moreover, a review of demand studies for Extra Virgin Olive Oil (EVOO) [12] found that after brand, organic production and origin certifications have the greatest influence on consumers’ willingness to pay (WTP). As brand creation is a lengthy and costly process, a higher sales price can be more easily achieved via these labelling schemes.

Applications that identify consumer preferences or intention to purchase for the product studied here, EVOO, exist for both the PDO (i.e., [11,12,13,14]) and organic production labels (i.e., [15,16]). Studies focusing on both labels simultaneously are less common. Other research [17,18,19,20,21,22,23,24] analyzed the consumption of other olive oils types.

The goal of this paper was to investigate how the two labels (geographical origin and production method) influence consumer food choices providing a significant contribution to our understanding of consumer preference for multiple product labelling. We focused on EVOO in Spain and two European food quality labels, those of PDO and the EU organic production. Willingness to pay for these labels was estimated using a stated preference valuation method, discrete choice experiment (DCE). In particular, we estimated whether consumers are willing to pay a premium for these EU labels and whether these can be seen as complementary or substitute strategies. The empirical analysis has been applied to the PDO “Aceite del Bajo Aragón”, a PDO olive oil produced in the province (NUTS3) of Teruel (Aragon), a rural territory with extreme climatic conditions, low productivity soils, extensive arid areas characterized by strong north-eastern winds, scarce and irregular rainfall and one of the lowest population densities in Spain. This area accounts for a total of 14,800 square kilometers and a population of 136,000 inhabitants with a population density of nine inhabitants per square kilometer, close to what is defined as a demographic desert. Moreover, if we subtract the population of the capital city (approximately 36,000), this density falls to under seven inhabitants per square kilometer. In addition, the region is characterized by an ageing population (46.5 years compared to 43.4 for the Spanish average) and follows a declining trend since the 1970s [25]. Teruel is considered a remote rural area that faces serious challenges to ensure its future economic development [26]. Agriculture continues to play an important role in its economy and most crops are rain-fed, with olive groves occupying 24,245 hectares in 2017 [25].

## 2. Materials and Methods

To achieve the objectives stated above, we used a DCE approach because this method allows the simultaneous valuation of multiple food attributes, is consistent with the random utility theory [27] and the choice task mimics real purchases in the market [28]. Consumers face different products described as combinations of different levels of attributes and must decide which of the available alternative products to buy or decide not to buy any of them.

### 2.1. Data Collection

Data were obtained from a self-administrated questionnaire delivered to a sample of individuals over 18 years of age resident in Aragon and responsible for food purchase in the household. The questionnaire was structured in three parts. The first part included questions related to olive oil purchasing and consumption habits. The second contained the DCE tasks, preceded by a description of the attributes and levels to participants to ensure that they were aware of the alternative products to be selected (see below). In addition, a cheap talk script [29] was included to encourage respondents to reveal their real preferences in order to minimize the possible hypothetical bias. The third part of the questionnaire contained socio-demographic characteristics and other features of the interviewees. Before administering the final questionnaire, a pilot survey was carried out with a small sample (N = 20) to check the correct understanding of the questionnaire and that the duration did not lead to fatigue effects.

The fieldwork took place in 2014 and the first interviewees were randomly selected from a consumer panel. They were then contacted via e-mail with the questionnaire (as an attachment or as a link for on-line completion) asking them to respond to the questionnaire and send it back. Furthermore, the questionnaire was provided to contact people in consumer associations and public city centers already known to us for their random distribution with a similar request for completion and return. The final sample used consisted of 540 valid observations representing a sample error of 4.2 % using a confidence level of 95% and the more conservative sample proportions (p = q = 0.5).

### 2.2. Choice Experiment Design

The first aspect of the choice experiment design that needs to be tackled is that of the selection of the attributes and their levels. Our final design included three attributes to respond to our research objective: two attributes reflecting the labels of interest and a price vector to allow the estimation of the willingness to pay. The levels of the attributes were firstly based on market research of the available olive oils in different shopping outlets (supermarkets and hypermarkets), which resulted in a database of 260 EVOO with information on price, bottle size, bottle type, presence of PDO and organic production labels and geographical origin. They were further based on the results of a focus group conducted with 12 (three men and nine women) olive oil shoppers in Zaragoza (the main town in Aragon) in order to examine the consumption habits of olive oil and the importance of several attributes. An interview guide was provided to help the moderator define the development of the meeting and articulate and organize various issues of the debate while respecting the time limit of 1 h. The guide identified three parts for the meeting: (i) an introduction with presentation of our study and distribution of some relevant information regarding PDO and Organic labels; (ii) a section with open, neutral and easy to understand questions; and iii) final summary of the discussions.

The analysis of the database on olive oils characteristics combined with the results from the focus group served to identify the most prevalent and purchased format of olive oil: one liter bottle of EVOO. In addition, the most important attributes for olive oil purchase were identified: price, brand, geographic origin indication in all its various specifications (PDO and other non-regulated claims such as local origin) and its production system (organic or conventional). Thus, the three attributes selected were price, geographic origin and organic production labels. Brand was discarded because, although the olive oil market is generally driven by brand, the market for EVOO with quality labels is not. This is because these high-quality olive oils are produced by small regional companies that focus more on the quality claimed on the label rather than the promotion of their brands. The levels of the attributes were set to two (presence, absence) for the two types of labels and to four for prices. Based on the information contained in the database we identified the highest and lowest price for one-liter bottles of EVOO: 3 € and 9 €, respectively. The other two price levels were taken to allow an even distribution of the price vector between the extremes (5 € and 7 €). As this research was funded by the Research Program of the Teruel Investment Fund we focused on the only PDO for olive oil in Teruel, “Aceite del Bajo Aragón”. The attributes selected and their levels are summarized in Table 1.

**Figure 1:**
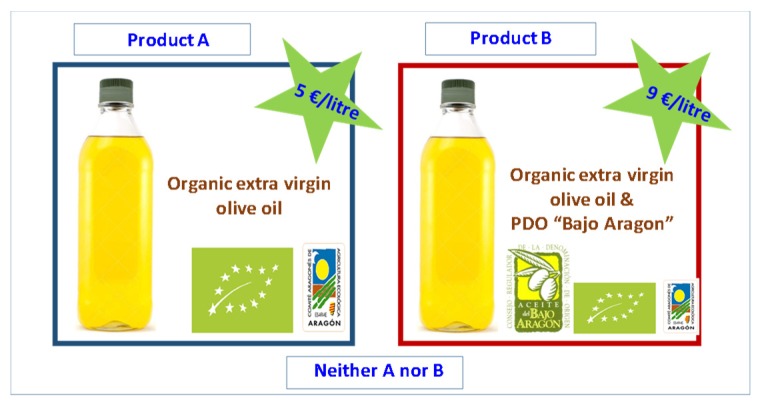
Choice set example of the unlabeled experiment *. * Original figures were presented in Spanish, Source: own elaboration.

A full factorial design for choice sets with two options plus a non-purchase alternative for three attributes with four, two and two levels, respectively, would lead to 256 choice sets [(4 × 2 × 2)^2^]. As these are far too many choices to present to our sample, we built the choice set design following the [30] theoretical framework to produce optimal and near-optimal designs for different number of alternatives, attributes and levels assuming zero priors [31]. Starting with the 16 profiles that represent the full factorial design to ensure that effects are uncorrelated, we applied a shifting procedure to these 16 profiles to create the other alternative. In particular, different generators (set of numbers that are applied to the starting design to shift the levels on the attributes based on orthogonal arrays) have to be used depending on the type of choice experiment to be designed [32]. The resulting design consisted of 24 choice sets. To avoid that interviewees must respond to a large number of choice sets and to minimize the risk of a fatigue effect, the total number of choice sets was randomly split into three blocks of eight choices. Respondents were randomly allocated to one of the blocks. Respondents should indicate which of the two products in each of the choice sets would choose, or whether they would not choose any of them. Figure 1 shows one example of a choice set.

### 2.3. Specification and Estimation

Choice modelling assumes that consumers choose between different products consisting of some attributes with different levels to maximize their utility. Following [33], total consumer utility of the product is the sum of the utilities for the different attributes. The consumer knows his utility but the researcher can only observe part and assume the rest behaves stochastically, following the random utility theory [27]. With this in mind, utility is assumed as a random variable that can be represented, for our empirical model, as follows:

(1) $$U_{njt}=\alpha +\beta_{1}PRICE_{njt}\;+\;\beta_{2}PDO_{njt}\;+\;\beta_{3}ORG_{njt}+\beta_{4}PDO*ORG_{njt}+\varepsilon_{njt}$$

where *n* is the number of respondents, *j* represents the different alternatives seen by the individual when making choices (two products plus the non-purchase option) and *t* the number of the choice set (in this case the eight choices made by the individual). Coefficient α is a constant reflecting the utility associated to the purchase of one of the alternatives coded as a dummy variable that takes the value of 0 for the non-purchase option (neither A nor B) and 1 for the two product alternatives (A and B). It is expected to be positive and statistically significant. PRICE is defined by the price levels in the design (3 €/L, 5 €/L, 7 €/L and 9 €/L). PDO and ORG are defined as dummy variables, where 1 indicates that the product carries the PDO and the organic labels, respectively and 0 otherwise. The interaction between the PDO and the organic labels (PDO*ORG) is calculated by multiplying PDO and ORG. Finally, ε_njt_ is an independent identically distributed (i.i.d.) error term over time, respondents and alternatives. 

If we assume that consumers are homogeneous in terms of preferences, the utility function expressed in (1) can be estimated using a conditional logit model (MNL) [27]. However, significant evidence (including the studies referenced in the introduction) have signaled that consumers’ preferences for food products are heterogeneous. To capture this, the model specification should allow the parameters to vary among individuals. Two alternatives exist to take into account preference heterogeneity: the Random Parameter Logit (RPL) model and the Latent Class logit (LC) model, depending whether preferences are assumed to be individual-specific or lumpy by consumer groups [34]. We do not have *a-priori* reasons to believe that preferences for these labels are lumpy and thus we implement an RPL model where each individual has his own specific preferences. Heterogeneity is included by adding a vector of parameters that incorporates individual preference deviations with respect to the mean preference values *β*_n_ in Equation (1). Moreover, correlations across utilities and across parameters could exist and appropriate specification of those correlations should take into account.

Correlation across utilities can be generated because the non-purchase alternative is experienced by the consumer in a real shopping situation, while the experimental alternatives are designed and vary across choice tasks. Therefore, the utilities of the designed options might be more correlated between them and have higher variance than the utilities of the non-purchase alternative. In other words, the experimental designed alternatives could share an extra error component that is not present in the utility of the experienced one [35]. To take into account the extra variance of experimentally designed alternatives, an additional error component must be included in the model specification. This results in the so-called Error Component Random Parameter Logit (ECRPL), which is parsimonious (it only requires one parameter extra) and improves the model fit [36]. In addition, correlation across parameters can be expected if some attributes are inter-dependent. In this case, the correlation structure of *β*_n_ should follow a multivariate normal distribution (normal with vector mean μ and variance–covariance matrix Ω). If at least some of the estimates for elements of the Cholesky matrix C (where C’C = Ω) are statistically significant, this means that dependence across parameters exists [37].

We report estimates of three models to identify the impact of the different econometric options on results and test whether the assumptions above hold. We start with a simple Multinomial Logit Model (MNL) assuming homogenous preferences. Second, we relax the homogeneity assumptions by estimating an RPL using the panel structure of the data and taking into account that each individual made eight choices [38]. Last, we allow for the existence of a correlation across utilities and parameters estimating an Error Component Random Parameters Logit (ECRPL) with correlated errors. All estimations were conducted using NLOGIT 5.0 assuming that price is a fixed coefficient (preferences for income are homogenous across consumers) and that the coefficients for the three dummy variables (PDO, ORG and PDO*ORG) are random following a normal distribution. For the estimation of the RPL and ECRPL models, 200 Halton draws rather than pseudo-random draws were used since the former provides more accurate simulations [38].

Expected signs for the estimated parameters relate to the underlying consumer theory and prior findings. α is expected to be positive and significant, indicating that consumers obtain a lower level of utility when they select the non-purchase option, as they are frequent consumers of olive oil. The PRICE coefficient is expected to have a negative sign as following a specific purchase decision, more disposable income should be preferred against less. Last, the PDO and ORG variables were expected to have a positive effect based on the evidence identified in the reviewed studies. We have no *a-priori* expectation for the interaction coefficient. If the estimated interaction coefficient is negative, both labels can be considered substitutes as the utility derived from the joint provision of them is lower than the sum of the utilities associated with the PDO and ORG considered in isolation. Therefore, some of the utility provided by each of the labels is already being provided by the other [39]. On the contrary, if this interaction coefficient is positive, both labels would be complementary as the presence of both labels adds additional utility to that provided by both in isolation.

Based on the parameters estimates, the relative importance for each of the attribute levels can be assessed. The importance scores (IS) were calculated by multiplying the absolute value of the estimated coefficients by the difference between the highest and lowest value of each attribute level [40]. The score measures the extent to which consumers’ utility changes as the level of the attribute is moved and is calculated using the following expression:

(2) $${\mathrm{IS}}_{1}\;=\;\frac{\beta_{1}\;\left(\mathrm{Highest}-\mathrm{Lowest}\right)}{\sum_{n}\beta_{k}\;\left(\mathrm{Highest}-\mathrm{Lowest}\right)}$$

where k indicates the number of attribute levels, in our case 4 (Equation (1)). IS provides the consumers’ importance ranking for the different attributes-levels (PRICE, PDO, ORG and PDO*ORG).

Finally, from the estimated parameters, the marginal willingness to pay (WTP) for each of the attributes and for the interaction between the PDO and the organic labels and the total marginal WTPs for the combination of the two labels including the interaction factor term were calculated. These WTPs are the extra price consumers are willing to pay for each of the labels. The marginal WTP is the price change associated with an increase in a given attribute and can be calculated as the ratio of the partial derivative of the utility function with respect to the non-monetary attribute of interest, divided by the derivative of the utility function with respect to the monetary one (price). Since in our empirical application the non-monetary attributes are coded as dummy variables, the marginal WTPs for the main effects are calculated by taking the ratio of the mean parameter estimated for the non-monetary attributes to the mean price parameter multiplied by minus one as follows:

(3) $${\mathrm{WTP}}_{2}\;=\;-\;\frac{\beta_{2}}{\beta_{1}}$$

In addition, the total marginal WTP, accounting for both main and interaction effects, are calculated as follows [41]:

(4) $${\mathrm{WTP}}_{\mathrm{Interaction}}\;=\;-\;\frac{(\beta_{2}+\beta_{3}+\beta_{4)}}{\beta_{1}}$$

## 3. Results and Discussions

### 3.1. Sample Descriptive Statistics

Table 2 presents the variables and characteristics of the sample and that of the target population (Aragon) and that of Spain. Women accounted for 65.1% of respondents above their weight in the reference populations, a value higher than the population averages but reflecting that women are still mainly in charge of food purchases in the household. The average age of those surveyed was 48.9 years and the average household size was 2.8 persons. These values are also slightly higher than the average values for Aragon and Spain, something that is logical for the latter since those under 18 were excluded from the sample. Regarding education, 55.7% of the respondents had a university degree and only 14.4% of them have primary studies. The greater proportion of people with university studies in the sample is common in this type of studies because more educated people are more prone to respond to questionnaires [42].

### 3.2. Estimation Results

The estimation results for the three models are shown in Table 3. The first column presents the results for the MNL, the second column for the RPL and the third shows the estimations for the ECRPL with correlated errors.

We can confirm that consumer preferences are heterogeneous according to the coefficients of the standard deviations of parameter distribution in the RPL model. Two of them are statistically significant at the 1% significance level, indicating that consumer heterogeneity exists. This is further confirmed by the improvement of the log-likelihood value at convergence and the adjusted pseudo R^2^. To calculate the gain in this model performance in the presence of the set of estimated parameters for the attributes levels, we compared the log likelihood of the model at convergence for the RPL (−3018.05) and the null log likelihood (−4317.63). In addition, to test the overall statistical significance of the model, we calculated the Likelihood Ratio (LR) between the log likelihood of the model at convergence and the restricted log likelihood (−4746), which accounts for 3456. This value is greater than the χ^2^ for 8 degrees of freedom [43], corroborating the overall significance of the model. To test whether utilities and parameters are indeed correlated we focus on the comparison between the RPL and the ECRPL models. Again, both the log-likelihood value at convergence and the adjusted pseudo R^2^ improve, indicating that the ECRPL model was better than the RPL. Moreover, the σ_ε_ for the error component was statistically significant, consistent with the idea that an error component model must be specified. To see whether parameters are correlated, we observe that two of the values in the diagonal of the Cholesky matrix were statistically significant at the 5% level, indicating that the errors were correlated, and thus, a multivariate normal distribution was the best assumption. In the same way, to calculate the gain in performance of the later model with the set of estimated parameters for the attributes levels, we compared the log likelihood of the model at convergence for the ECRPL (−2802.35) and the null log likelihood (−4317.63). In addition, to test the overall statistical significance of the model, we calculated the Likelihood Ratio (LR) between the log likelihood of the model at convergence and the restricted log likelihood (−4746), which accounts for 3888. This value is greater than the χ^2^ for 12 degrees of freedom corroborating the overall significance of the model. We selected the ECRPL with correlated errors as the best specification; therefore, this model was used to provide results and to conduct further analysis.

As expected, α was positive and significant, indicating that consumers obtain higher utility from choosing any alternative than from the non-purchase option. The PRICE variable was negative and statistically significant in line also with theoretical consistent expectations. The estimated parameters for the main effects of the PDO and ORG labels were positive and statistically significant at the 1% level. Our consumers positively value the PDO and the organic production labels, and the effect of the PDO label in the utility is higher than the effect of the organic label. Last, the interaction coefficient between the two labels was negative and statistically significant. This result indicates that the utility for the olive oil with both the PDO and the organic production labels is slightly and negatively adjusted when compared to the sum of the utilities derived by the PDO and the organic production labels. Thus, both labels can be considered substitutes indicating an overlap between the concepts associated to each of the labels. In other words, the concepts are perceived as interchangeable indicating that no added value is created by the combination of the two labels [44].

Table 4 presents the relative importance of the two labels and the price (Equation (2)) as well as the marginal WTP for the main effects (Equation (3)) and the total WTP accounting for the main and interaction effects (Equation (4)). The importance scores indicate that the most important attribute when purchasing EVOO is PRICE, which drives 64% of the final choice. The PDO label and the EU organic logo are secondary attributes, with PDO explaining twice the choice of the organic. This result is in agreement with previous empirical papers mentioned above and in the literature review by [12]. In addition, [18] found that for a majority of consumers the most important attribute for the EVOO is the price.

Marginal WTP estimates for each of the labels were positive and statically significant. This result is in agreement with several empirical applications on olive oil [17,18,19,20] that also estimate positive WTPs for these two labels. On the contrary, [22,23] found a negative valuation for organic olive oil in Spain and [21] state that Italian consumers are not willing to pay for the PDO label. In addition, our findings indicated that consumers’ valuation of the PDO label was higher than that of the organic production label, in particular, the extra price consumers were willing to pay is double for the PDO label. Specifically, consumers were willing to pay an extra premium of approximately 2 €/L for a bottle with the PDO label in respect to one without this label, and approximately 1 €/L for a bottle with the organic label in relation to one without this label. This finding is in line with [17], who found that the WTP for the PDO is slightly higher than the WTP for the organic label. However, our results differ from [19,20], who estimated that the WTP for the organic label is higher than the WTP for the PDO. The marginal WTP for the interaction (PDO*ORG) is negative (−0.377 €/L), indicating the price discount when the two labels are presented together [45]. Thus, the total WTP accounting for the main and interaction effects was 2.6 €/L instead of 3 €/L. If the EVOO is labelled with the PDO and organic label, a typical consumer is willing to pay 2.6 €/L more than for the EVOO without these two labels. However, the value of a single label of PDO is 2 €/L and that of organic is 1 €/L [46]. Consumers might perceive the values of the PDO and organic labels to be overlapping when they are presented simultaneously [45]. Then, our data show that, on average, consumers find that for EVOO the PDO and organic labels are competing because either some of the quality aspects of each label are partly covered by the other or that information overload generates mistrust. However, because preferences were heterogeneous, we found a minority of our sample (12%) who had a positive WTP for the interaction of both labels. Thus, we turn to examining who these consumers are.

To achieve this, we grouped the respondents into two segments according to whether the coefficient of the interaction between the two labels in the ECRPL model with correlated errors $\left(\beta_{4}\right)$ has a negative or positive impact on utility. Then, we looked at whether there were differences between those segments based on socio-demographic and economic characteristics and some olive oil shopping habits (frequency of food shopping, importance attached when shopping olive oil and EVOO to different aspects, purchase intent of EVOO with PDO and actual purchase of EVOO with PDO and/or organic label). These tests of differences between the two segments were done using chi-square or analysis of variance test [47], for discrete and continuous variables, respectively. Table 5 presents the analysis of variance and chi-square test results between the two segments and the different consumer characteristics that will allow profiling them.

In particular, some of the consumers’ characteristics displayed in Table 2 and the olive oil shopping habits mentioned above were found to be statistically different between segments at least at a 10% significance level (Table 5). Thus, whether considering the two labels substitutes or complements is explained by differences in some consumer socio-demographics, purchasing habits and attitudes. In particular, the segment that considered both labels complementary (thus providing a higher value to the multiple presence of labels in the product) consists of consumers with university level education, living in smaller households, even if their monthly income is lower. In addition, the frequency of food purchase has been found statistically different and consumers in the segment that considered both labels as complementary are more involved in the food purchasing of the household. As far as the importance consumers attached to different aspects while shopping for olive oil and EVOO, they considered both labels complementary and gave more importance to organic production and taste when shopping for olive oil, and to the olive variety (“empeltre”) and the type of bottle when shopping for EVOO, than those that considered them substitutes. Thus, knowledge of the specific characteristics of the PDO (at least an 80% of “empeltre” variety has to be used if the PDO “Aceite del Bajo Aragón” is to be used) means consumers perceive the presence of both labels as complementary. On the contrary, they gave less importance to the taste of the EVOO. Consumers who considered both labels substitutes are more likely to buy EVOO with the PDO “Aceite del Bajo Aragón”. Finally, a higher proportion of consumers that considered both labels substitutes had sometimes purchased EVOO with the PDO and/or organic label.

## 4. Conclusions

In this paper we have assessed how EU food quality labels, in particular PDO and organic labels, influence consumer food choice. The results of the DCE application confirm that consumers derive positive utility for EVOO carrying these EU quality labels. In addition, while both PDO and organic production labels are positively valued by consumers, the utility for the olive oil with both the PDO and the organic production labels is slightly and negatively adjusted when compared to the sum of the utilities derived by the PDO and the organic production.

Consumers might perceive the values of the PDO and organic labels to be overlapping when they are presented simultaneously. Our data shows that for the majority of consumers, the PDO and organic labels in the EVOO are competing (substitutes), while a small group of consumers considered them as complementary.

The finding can help producers identify an optimal labelling strategy to maximize returns on certification investments. As the extra price consumers are willing to pay for the PDO label is double (2 €/L) that for the organic one (1 €/L), producers should first apply the PDO label. In a second stage, they could evaluate the appropriateness of adding an organic label to the previous PDO taking into account that the joint provision of both will not lead to the full 1 €/L price premium for the organic label, but only an extra price of 0.6 €/L over the price of the PDO EVOO. Thus, if the costs of applying the ORG label exceed this price, it is advisable to use only the PDO label. Assessing the profitability of organic conversion based only on the WTP for the stand-alone label could lead to sub-optimal outcomes.

This work provides a new contribution to the debate on whether two different EU quality labels, the PDO and the organic, are most valued by consumers and it is among the first to provide an answer to the question of whether the PDO and the organic labels are complements or substitutes.

Although price is the biggest determinant of food consumption in the EU, a fact confirmed by our EVOO consumers, consumers prefer and value products with one of the PDO or organic labels, but there is a diminishing marginal utility of additional labels. As EU labels are a driving force for demand for foods labelled as such, we recommend public authorities and producing and marketing companies promote and implement promotional campaigns to increase awareness of EU labels. This would likely increase the demand for this type of food and should be directed above all at young people, as they are most concerned with issues of food quality, safety and sustainability.

Finally, this work has some limitations that must be taken into account and that constitute other avenues of research for the future. The result of this study cannot be automatically transferred to other product categories. The sample used was not sufficiently representative and the choice experiment could be further extended including other products and attributes. The study has been carried out solely for a PDO for olive oil and in a Spanish region, thus, the empirical results are reduced only to this geographical coverage. With 29 PDOs for olive oil in Spain and located in a small number of regions, it would be appropriate to extend the case studies that would allow these results to be contrasted and generalized. Furthermore, although [48] found that hypothetical bias was not high when analyzing the consumer valuation for two claims, local and organic, for tomatoes in the US, the use of a real choice experiment would be advisable to avoid hypothetical bias and further corroborate the findings.

## Figures and Tables

**Table 1:** Extra virgin olive oil attributes and levels.

Attribute	Level	Visual Logo
Price (€/L)	3 €/L 5 €/L 7 €/L 9 €/L	Price per liter tag
Geographic origin “Aceite del Bajo Aragón”	Presence Absence	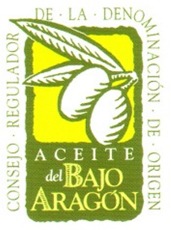
Organic production	Presence Absence	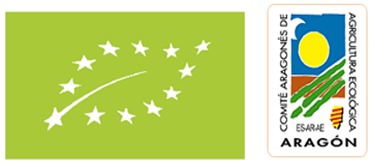

Source: own elaboration.

**Table 2:** Socioeconomic variables of the sample, Aragon and Spanish population.

Variable	Definition	Sample	Aragon	Spain
Gender	Female (dummy) (%)	65.1	50.3	50.8
Age (sample average)	Age (continuous)	48.9	43.9	42.1
Household size (average)	Number of people	2.8	2.5	2.5
Household monthly *per capita* income	Income (Above sample average) (%)	46.4	n.d.	n.d.
Highest level of education achieved	Primary studies (%)	14.4	27.2	25.5
	Secondary studies (%)	29.8	46.1	46.3
	University degree (%)	55.7	26.6	28.1

Source: Own elaboration based on data from the questionnaire and [25].

**Table 3:** Estimates for the utility models.

	MNL	RPL	ECRPL
Mean Values			
α	3.2314 (36.07) ***	4.098 (34.53) ***	5.4675 (29.70) ***
PRICE	−0.4919 (−37.10) ***	−0.6216 (−34.49) ***	−0.6634 (−52.11) ***
PDO	0.9442 (11.89) ***	1.2636 (11.68) ***	1.3111 (11.43) ***
ORG	0.5815 (8.86) ***	0.6537 (6.77) ***	0.6819 (6.73) ***
PDO*ORG	−0.2947 (−3.50) ***	−0.2874 (−2.92) ***	−0.2501 (−1.99) ***
Standard deviations of parameter distributions			
PDO	–	1.3480 (13.75) ***	1.4103 (8.87) ***
ORG	–	1.4200 (16.41) ***	1.2713 (11.57) ***
PDO*ORG	–	0.0555 (0.33)	0.4490 (2.96) ***
Diagonal values in Cholesky matrix			
PDO	–	–	1.4103 (8.87) ***
ORG	–	–	1.0757 (9.88) ***
PDO*ORG	–	–	0.0011 (0.00)
Standard deviation of the latent random effect			
σ_ε_		–	2.4062 (13.87) ***
N	4320	4320	4320
Log-likelihood at convergence	−3238.1	−3018.0	−2802.3
Adjusted Pseudo R^2^	0.25	0.36	0.41

Source: own elaboration. Wald statistics are in parenthesis. Null log likelihood = −4317.6. Restricted log likelihood = −4746.0. *** indicate significance at 1%.

**Table 4:** Relative importance and marginal willingness to pay for the attributes (%, €/L).

	Importance Score	Marginal WTP Main Effects	Marginal WTP Total: Main and Interaction Effects
$\beta_{1}:$PRICE	64.0		
$\beta_{2}:\;$PDO	20.9	1.9765 ***	
$\beta_{3}:\;$ORG	10.9	1.0275 ***	
$\beta_{4}:\;$PDO*ORG	4.2	−0.3770 ***	2.6

Source: own elaboration. ***, ** and * indicate significance at 1%, 5% and 10%, respectively.

**Table 5:** Characterization of consumers’ segments.

Characteristics	Complement Label	Substitute Labels	Total Sample
Proportion of respondents in the segments	12%	88%	
Highest level of education achieved*			
Primary	17.9	13.4	14.4
Secondary	19.4	31.3	29.8
University	62.7	54.7	55.7
Household monthly income (average) **	2284	2570	2534
Household size (average) **	2.6	2.9	2.8
Frequency of food purchase*			
Always	58.2	45.0	46.7
Often	25.4	34.3	33.1
Sometimes	16.4	16.3	16.3
Hardly never	0.0	4.4	3.9
Importance attached when shopping olive oil to:			
Organic production **	2.8	2.4	2.5
Taste *	4.3	4.1	4.2
Purchases EVOO with PDO label at least sometimes * (%)—Yes	68.7	77.6	76.5
Purchase organic EVOO at least sometimes * (%)—Yes	68.7	76.3	75.4
Intention to purchase EVOO with the PDO “Aceite del Bajo Aragón”			
Yes and likely yes	68.7	79.3	77.9
I do not know	22.4	15.9	16.7
No and likely no	8.9	4.9	5.4
Importance attached when shopping EVOO to:			
Taste	4.0	4.2	4.2
Variety (Empeltre)	3.4	3.1	3.1
Type of bottle (plastic, glass, etc.)	3.2	2.9	2.9

Source: own elaboration. Note: ***, ** and * denotes statistical significance at 1%, 5% and 10% respectively.
